# Supplementary material for: Risk Assessment During Nest Defense Against Three Simulated Predators by Female Northern House Wrens ( Troglodytes aedon )
Source: Ecol Evol. 2025 Jan 31;15(2):e70942. doi: 10.1002/ece3.70942 (PMC11783239; doi:10.1002/ece3.70942)
Supplement: Supplementary file 1 — Tables S1–S6. [file ECE3-15-e70942-s001.docx]

**Supplementary Materials**

Statement on STRANGE framework

Studies of animal behavior can be misinterpreted due to sampling biases, and the STRANGE framework provides a method for identifying these potential issues (Webster and Rutz 2020). All subjects in this study were free-living songbirds, and we included all individuals that nested on our study sites regardless of age or nesting location, making it unlikely that social background biased our results. As detailed above, a small number of individuals were excluded, namely those that failed to respond within 7 minutes during Experiment 1 before our protocol was adjusted to be more inclusive or those that responded so forcefully that they knocked the predator decoy from the box. Thus, we may have failed to sample a subset of both low and high responders. Although this approach may have biased our sample towards intermediate responders, we think this potential bias is unlikely to have skewed our results for a few reasons. First, our sample size is large for a field study and it includes individuals that responded both aggressively and weakly to the predator decoys, so it is unlikely that we failed to sample the extremes of behavioral variation in this population. Second, we conducted repeated-measures sampling, which required subjects to be excluded from all treatments rather than only those trials where we were unable to collect data. This approach limits the likelihood of bias as each individual effectively serves as its own control. With respect to seasonal changes in behavior, we sampled evenly across the entire breeding season and included measurement date as a covariate in our models to test for this effect. Because each subject experienced two sampling events it is possible that habituation affected responses to the second treatment. We limited this effect by presenting trials on separate days in a counterbalanced order and included treatment order in our models to test for an effect of habituation.

It is also possible that carryover effects caused by sampling the same individuals between years could bias the behavioral responses in Experiment 2. We think that this outcome is unlikely given the length of time between the two experiments, but if the same females were sampled in both years carryover effects remain possible. It is difficult to determine how many females were sampled in both experiments because only 13 of 47 females (27.7%) were banded in experiment one, including six females banded in previous breeding seasons. Of those banded females, only one (7.7.% return rate) was definitely sampled in experiment two. It is likely that some of the unbanded females also returned to be sampled again, and some inferences can be drawn from males, which were banded at much higher rates. In experiment one, 43 of 47 males (91.5%) were banded and eight males returned to be sampled again in experiment two (18.5% return rate). Based on these data, it seems likely that 10-20% of females were sampled in both experiments.

*Table S1 - Locations of field sites and number of nest boxes present.*

| Site | Location | Number of Boxes |
| --- | --- | --- |
| Big Walnut Elementary School, Sunbury, Ohio | 40°14'02.6"N  82°51'53.1"W | 15 |
| Big Walnut High School, Sunbury, Ohio | 40°14'00.9"N  82°51'37.3"W | 41 |
| Davis Family Property, Sunbury, Ohio | 40°15'50.2"N  82°49'52.8"W | 36 |
| Fink Family Property, Marysville, Ohio | 40°16'42.4"N  83°15'43.3"W | 21 |
| Kraus Research Preserve, Delaware, Ohio | 40°11'45.8"N  83°03'47.1"W | 42 |
| Ohio Wesleyan University and nearby homes, Delaware, Ohio | 40°17'47.7"N  83°03'53.8"W | 25 |

*Table S2 - Summary of the number of trials that include an observation of time spent in or on the next box. Experiment 1 tested responses to a simulated snake and chipmunk. Experiment 2 tested responses to a simulated snake and hawk.*

| Experiment | Time spent in the nest box | Time spent on the nest box |
| --- | --- | --- |
| 1 | 4/76 (5.3%) | 12/76 (15.8%)^1^ |
| 2 | 4/76 (5.3%) | 10/76 (13.2%)^2^ |

^1^9 of 12 observations were for 10 seconds or less (75%)

^2^7 of 10 observations were for 10 seconds or less (70%)

*Table S3 – Analysis of model fixed effects and covariates for Experiment 1. *P<0.05*

|  | Day of the Year | | Male Presence | | Order | | Interaction with Order | |
| --- | --- | --- | --- | --- | --- | --- | --- | --- |
| Behavior | *F*, *Χ^2^* or *z* | *P* | *F*, *Χ^2^* or *z* | *P* | *F*, *Χ^2^* or *z* | *P* | *F*, *Χ^2^* or *z* | *P* |
| Hits | -1.00 | 0.32 | -0.32 | 0.75 | -1.00 | 0.32 | 0.96 | 0.34 |
| Flyovers | -0.08 | 0.94 | 1.38 | 0.17 | -1.46 | 0.14 | 1.42 | 0.16 |
| Time within 5 m | 0.33 | 0.57 | 4.20 | 0.048* | 0.004 | 0.95 | 2.30 | 0.14 |
| Closest Approach | 0.31 | 0.58 | 4.22 | 0.048* | 1.33 | 0.26 | 1.85 | 0.18 |
| Latency to Return | 8.87 | 0.006* | 7.86 | 0.009* | 1.59 | 0.23 | 0.67 | 0.42 |
| Alarm Calls | 0.18 | 0.67 | 1.01 | 0.32 | 0.10 | 0.76 | <0.001 | 0.99 |

*Table S4 – Analysis of model fixed effects and covariates for Experiment 2.*

|  | Day of the Year | | Male Presence | | Order | | Interaction with Order | |
| --- | --- | --- | --- | --- | --- | --- | --- | --- |
| Behavior | *Χ^2^* or *F* | *P* | *Χ^2^* or *F* | *P* | *Χ^2^* or *F* | *P* | *Χ^2^* or *F* | *P* |
| Time within 5 m | 2.16 | 0.15 | 0.54 | 0.47 | 0.02 | 0.89 | 0.47 | 0.50 |
| Closest Approach | 1.16 | 0.29 | 1.15 | 0.29 | 0.08 | 0.78 | 0.59 | 0.45 |
| Latency to Return | 1.06 | 0.31 | 0.10 | 0.75 | <0.001 | 0.98 | 0.001 | 0.97 |
| Alarm Calls | 0.13 | 0.71 | 0.07 | 0.80 | 0.10 | 0.75 | 0.11 | 074 |

*Table S5 - Results of nonparametric analysis of Experiment 1 behavior.*

|  | First Trial | | Second Trial | |
| --- | --- | --- | --- | --- |
| Behavior | *W* | *P* | *W* | *P* |
| Hits | 200 | 0.41 | 118 | 0.09 |
| Flyovers | 220.5 | 0.15 | 126.5 | 0.17 |
| Time within 5 m | 204 | 0.35 | 168 | 0.91 |
| Closest Approach | 147 | 0.39 | 210.5 | 0.23 |
| Latency to Return | 116 | 0.82 | 109 | 0.98 |

*Table S6 - Results of nonparametric analysis of Experiment 2 behavior. *P<0.05.*

|  | First Trial | | Second Trial | |
| --- | --- | --- | --- | --- |
| Behavior | *W* | *P* | *W* | *P* |
| Hits | 280 | <0.001* | 288 | <0.001* |
| Flyovers | 350 | <0.001* | 351 | <0.001* |
| Time within 5 m | 223 | 0.21 | 170 | 0.78 |
| Closest Approach | 4 | <0.001* | 8.5 | <0.001* |
| Latency to Return | 92 | 0.048* | 111.5 | 0.19 |
